# Concerning the Prevalence of HPV Genotypes and the Evaluation of Pap smear Results in Iranian Population: An Update

**DOI:** 10.30699/ijp.2021.523796.2578

**Published:** 2021-05-09

**Authors:** Sina Neshat, Padideh Daneii, Negar Neshat, Sina Raeisi, Saba Raeisi, Seyed Mohammad Malakooti, Noushin Afsharmoghaddam

**Affiliations:** 1 Student Research Committee, School of Medicine, Isfahan University of Medical Sciences, Iran; 2 Student Research Committee, School of Medicine, Iran University of Medical Sciences, Iran; 3 Pathology Department, Shahid Beheshti University of Medical Sciences, Tehran, Iran

Dear Editor

The authors read the exciting article by Mina Mobini Kesheh* et al.* "The Prevalence of HPV Genotypes in Iranian Population: An Update"(1). We are willing to share our study results that evaluated HPV genotypes and related Pap smear cytology findings in a similar but briefer way in a total of 1500 patients referred to a local pathology laboratory between years 2019-2020 in Esfahan, Iran. All of the patients were sexually active females aged 25-55. All patients who referred to the laboratory for testing were considered as the statistical society of the research and all males and patients with active illnesses were excluded from the study. Two samples were collected from each patient, one for Pap smear study and the other for polymerase chain reaction (PCR) test using DNA extracting kits (Bioneer™, South Korea) to detect different serotypes of human papillomavirus (HPV) infection.

In this study, 236 samples were reported positive for HPV infection. By examining the genotype of positive subjects, it was found out that 14.8% of those samples contained subtype 16, and 1.7% were infected with type 82 in HPV test reports, which are in agreement with the aforementioned article. It was observed in patients with uterine cancer (2), of which 22.2% were positive for papilloma 16 subtype. Meanwhile, studies have shown that the most common type of HPV in women with cervical cancer is type 16 (3). As we mentioned in the main article, more than 50% of these tests report false-negative results. In the current screening system, errors can occur while collecting the smears and analyzing (4, 5).

Thus Type 16 is the most serotype involving in HPV infections among the average Iranian population (2, 6, 7, 8). It appeared as the type of HPV infections with the most frequency in our study (14.8%) and seemed to have more severe exudates. All the specimens that have been proved by PCR assay to be infected with HPV were reported to include Anucleated squamous cells or Anucleated Keratinized squamous cells (*P*=0.009), which pathologically mostly lead to Squamous intraepithelial lesions (9). Our findings suggested which Pap smear report is most associated with each serotype of HPV; however, the "No Inflammation" state is the most common among all serotypes (39.4%). Detailed HPV PCR test and Pap smear cross-tabulation results are shown in . We recommend cytologists report the presence of Anucleated squamous or Keratinized cells and exudates in the positive smears in order to prevent HPV-related Cancers in the population.

**Table 1:** Cytology * PAP Cross-tabulation

HPV Serotypes	MILD	Moderate	Severe	LSIL	HSIL	ASCUS	No Inflammation	Total
16	6	3	7	2	1	1	15	35
18	5	1	2	0	0	2	9	19
31	6	4	3	2	0	0	5	20
35	1	0	0	0	0	1	4	6
39	5	1	3	1	0	2	5	17
45	3	2	1	0	0	0	3	9
51	1	1	3	2	0	1	7	15
52	3	2	7	2	0	1	10	25
53	2	2	5	0	0	2	7	18
56	4	2	2	2	0	0	6	16
58	3	2	3	0	0	2	5	15
59	0	0	1	0	0	0	4	5
66	1	0	1	2	0	0	4	8
68	4	4	3	0	0	1	5	17
73	1	2	1	0	0	0	3	7
82	1	0	0	1	1	0	1	4
Total	46	26	42	14	2	13	93	236

## References

[1] Gelgoot E, Caufield-Noll C, Chisolm M (2018). Using the visual arts to teach clinical excellence. Med Ed Publish..

[2] Mukunda N, Moghbeli N, Rizzo A, Niepold S, Bassett B, DeLisser HM (2019 ). Visual art instruction in medical education: a narrative review. Med Educ Online.

[3] Lippi D, Bianucci R, Donell S The visual arts and medical education.

[4] Agarwal G G, McNulty M, Santiago (2020). Impact of Visual Thinking Strategies (VTS) on the Analysis of Clinical Images: A Pre-Post Study of VTS in First-Year Medical Students. J Med Humanit.

[5] Poirier TI, Newman K, Ronald K (2020). An Exploratory Study Using Visual Thinking Strategies to Improve Undergraduate Students' Observational Skills. Am J Pharm Educ.

[6] Chacko TV (2018). Emerging pedagogies for effective adult learning: From andragogy to heutagogy. Arch Med and Health Sci.

[7] Muganga L, Ssenkusu P (2019). Teacher-Centered vs Student-Centered. Cultural and Pedagogical Inquiry.

[8] Serin H (2018). A comparison of teacher-centered and student-centered approaches in educational settings. Int J Soc Sci & Educ Studies.

